# Sorting lung tumor volumes from 4D‐MRI data using an automatic tumor‐based signal reduces stitching artifacts

**DOI:** 10.1002/acm2.14262

**Published:** 2024-01-17

**Authors:** Mark Warren, Alexander Barrett, Neeraj Bhalla, Michael Brada, Robert Chuter, David Cobben, Cynthia L. Eccles, Clare Hart, Ehab Ibrahim, Jamie McClelland, Marc Rea, Louise Turtle, John D. Fenwick

**Affiliations:** ^1^ School of Health Sciences, Institute of Population Health University of Liverpool Liverpool UK; ^2^ The Clatterbridge Cancer Centre NHS Foundation Trust Liverpool UK; ^3^ Molecular & Clinical Cancer Medicine, Institute of Institute of Systems, Molecular and Integrative Biology University of Liverpool Liverpool UK; ^4^ Christie Medical Physics and Engineering The Christie NHS Foundation Trust Manchester UK; ^5^ Division of Cancer Sciences, School of Medical Sciences, Faculty of Biology, Medicine and Health University of Manchester Manchester UK; ^6^ Department of Health Data Science, Institute of Population Health University of Liverpool Liverpool UK; ^7^ Radiotherapy The Christie NHS Foundation Trust Manchester UK; ^8^ Department of Medical Physics and Bioengineering University College London London UK

**Keywords:** 4D‐MRI, NSCLC, principal components, respiratory sorting, stitching artifacts

## Abstract

**Purpose:**

To investigate whether a novel signal derived from tumor motion allows more precise sorting of 4D‐magnetic resonance (4D‐MR) image data than do signals based on normal anatomy, reducing levels of stitching artifacts within sorted lung tumor volumes.

**Methods:**

(4D‐MRI) scans were collected for 10 lung cancer patients using a 2D T2‐weighted single‐shot turbo spin echo sequence, obtaining 25 repeat frames per image slice. For each slice, a tumor‐motion signal was generated using the first principal component of movement in the tumor neighborhood (TumorPC1). Signals were also generated from displacements of the diaphragm (DIA) and upper and lower chest wall (UCW/LCW) and from slice body area changes (BA). Pearson *r* coefficients of correlations between observed tumor movement and respiratory signals were determined. TumorPC1, DIA, and UCW signals were used to compile image stacks showing each patient's tumor volume in a respiratory phase. Unsorted image stacks were also built for comparison.

For each image stack, the presence of stitching artifacts was assessed by measuring the roughness of the compiled tumor surface according to a roughness metric (*Rg*). Statistical differences in weighted means of *Rg* between any two signals were determined using an exact permutation test.

**Results:**

The TumorPC1 signal was most strongly correlated with superior‐inferior tumor motion, and had significantly higher Pearson *r* values (median 0.86) than those determined for correlations of UCW, LCW, and BA with superior‐inferior tumor motion (*p* < 0.05).

Weighted means of ratios of *Rg* values in TumorPC1 image stacks to those in unsorted, UCW, and DIA stacks were 0.67, 0.69, and 0.71, all significantly favoring TumorPC1 (*p* = 0.02–0.05). For other pairs of signals, weighted mean ratios did not differ significantly from one.

**Conclusion:**

Tumor volumes were smoother in 3D image stacks compiled using the first principal component of tumor motion than in stacks compiled with signals based on normal anatomy.

## 1 INTRODUCTION

4D‐magnetic resonance imaging (4D‐MRI) can be used to localize lung tumors for planning of radiotherapy (RT). Routinely, though, tumors and their respiratory motion envelopes are localized using 4D‐computed tomography (CT) techniques. Compared to CT, MRI has superior soft tissue contrast,^1^, ^2^ and since MRI scanners do not deliver ionizing radiation, images can be collected over a longer period, potentially allowing tumor motion to be more fully characterized.^3^, ^4^, ^5^, ^6^, ^7^ Using modern integrated MR‐linacs, 4D‐MRI scans can be collected immediately before treatment for use in adaptive RT.^8^, ^9^, ^10^ Additionally, a few slices can be repeatedly imaged during radiation delivery enabling real‐time RT tumor tracking or gating without the need to use external markers.^11^

Currently, it is challenging to collect repeat high‐quality 3D MR images fast enough to directly visualize the whole tumor throughout the course of multiple breathing cycles.^5^, ^12^ As a result, respiratory‐correlated 4D‐MRI scanning is used, generating image data which is retrospectively sorted to show the whole tumor volume over several phases of a representative breathing cycle. A common method of generating respiratory‐correlated 4D‐MR images is to use a 2D‐readout, collecting complete *k*‐space data for a single 2D image at a time.^12^ Typically scanning continues over a few minutes, repeatedly cycling through a set of 2D slices. Then the slice images are retrospectively sorted into respiratory bins and stitched together to create a series of 3D images. The 2D‐readout method is easy to implement, provides a choice of contrasts (T1/T2, T2), and compared to using 3D‐readouts has much shorter reconstruction times of around 40 s.^9^, ^13^

A limitation of the 2D‐readout approach is the presence of stitching artifacts, which reduce the geometric accuracy of the resulting 3D tumor volumes. Stitching artifacts occur when neighboring 2D slices collected in different respiratory states are erroneously stitched together in a 3D image supposedly representing a single respiratory phase. This is caused by a lack of precision in the respiratory signal used in the frame sorting process.^14^ Respiratory signals are often generated from the displacement of normal tissue elements visible in the MR images, for example, the diaphragm or chest wall, or from external sources such as respiratory bellows.^15^ As such, they may not describe the precise respiratory state of the tumor at the time of image collection, since movement of one part of the thorax does not have a consistent relationship with movement of another.^16^

Further imprecision may be introduced during the signal generation process. For example, to avoid the time‐overhead involved in generating a signal by repeatedly collecting navigator images of the same tissue element throughout 4D‐MRI acquisition, so‐called “self‐sorting” signals have been built by standardizing and then merging slice‐specific signals extracted directly from each imaged slice.^17^ Methods of standardizing signal values from different slices are heuristic, and to date, there has been little exploration of the relative abilities of self‐sorting signals to stitch together tumor volumes.

This study examines the precision of several respiratory signals and determines which produces the best stitched‐together tumor volumes. Alongside slice‐specific signals derived from the diaphragm and body surface, we also examine a novel signal generated from movement in the tumor neighborhood. As this signal is predominantly influenced by the motion of the tumor rather than of normal tissues, we hypothesize that tumor volumes compiled using it will contain fewer or smaller stitching artifacts than do volumes compiled using signals derived from other locations.

## 2 METHODS

### 2.1 MRI acquisition

Ten patients with non‐small cell lung cancer (NSCLC) were imaged using a 1.5T Siemens MRI scanner up to 2 weeks prior to starting RT. During the imaging session, sagittal and coronal 4D‐MRI datasets were acquired using a 2D T2‐weighted single‐shot turbo spin echo (HASTE) sequence with a fat saturation pre‐pulse (144° flip angle, 52 ms echo time, 192 echo train length, 4.72 ms echo spacing, and 660 ms repetition time, 300 mm × 300 mm field of view). Slices typically covered a region extending from just above the lung apex to just below the diaphragm and were collected in voxels of dimensions 1.5 × 1.5 × 5 mm^3^ where the 5 mm dimension describes slice thickness. During the cycling acquisition, 15−20 contiguous slices were first collected 25 times in the sagittal imaging plane over a total scan time of 3.75−5 min. Slices were captured in an interleaved order, odd‐numbered first then even ones, to avoid cross‐talk and saturation effects and to lessen under‐sampling artifacts introduced into 4D scans by irregular breathing.^18^, ^19^ The acquisition was then repeated in the coronal imaging plane.

### 2.2 Signal generation

#### 2.2.1 Principal component signal of tumor displacement

**FIGURE 1:**
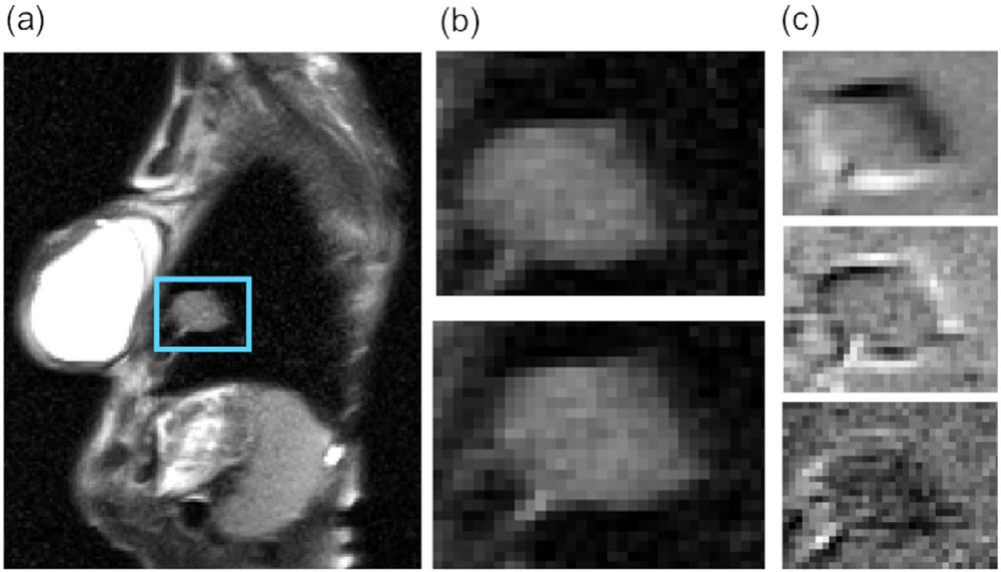
TumorPC1‐3 signal generation. (a) For each patient, a region of interest (ROI) (blue box) is drawn to encompass the tumor and its extent of motion in all sagittal image slices. (b) Locations of the tumor within the ROI at end‐inspiration and end‐expiration respectively. (c) The first three PCs of frame‐to‐frame variations of the image data within the ROI.

Our novel signal utilizes an automated but indirect measure of frame‐to‐frame tumor displacement. The only user action needed is to crop all slices to a region‐of‐interest (ROI) large enough to capture the full extent of the tumor and its movement in all frames across all slices, but small enough to exclude as much normal anatomy as possible. The aim is to define a region in which frame‐to‐frame variations reflect movement of the tumor, rather than of normal tissues. Such variations can be quickly and automatically described by a 2D principal components (PC) analysis of pixel intensities within the region (Figure 1).

For each repeat frame of a slice, the cropped image was transformed into a row vector of length *R*. The row vectors of the *N_f_* cropped frames collected per slice were then compiled into a matrix *M* of dimensions *N_f_* (column length) x *R* (row width), and *M* was centered by subtracting from each element the mean value of elements in its column, and scaled by dividing the centered elements in each column by their standard deviations.

A complete set of *N_f_* PCs was obtained from a singular value decomposition of the *M* matrix.^20^ For each repeat frame of a slice*, N_f_* signals were obtained from the projection of the frame's centered and scaled row vector onto the slice's set of PCs. Our analysis focused on only three of these signals, TumorPC1, TumorPC2, and TumorPC3, which describe the contributions of PCs 1−3 to the frame's cropped image. These three PCs accounted for 65% of frame‐to‐frame variance over all slices analyzed in the 10 patients. The TumorPC signals are slice‐specific in the first instance but, like signals built from displacements of the diaphragm or chest wall, they can be standardized and merged into self‐sorting signals collated from many slices. In this study we largely focus on their use as self‐sorting signals.

#### 2.2.2 Direct measures of tumor displacement

To quantify tumor motion, two direct measures of tumor displacement were also extracted from slices. The first described frame‐to‐frame changes in the displacement of the superior surface of the tumor. On each frame, the image intensity profile along a line passing superiorly‐inferiorly (SI) through the top surface of the tumor at its anterior‐posterior (AP) midpoint was calculated from pixel intensities along the line, averaged across three consecutive pixels in the AP direction. The SI position of the surface of the tumor, TumorSURF, was identified as the 50% point on an s‐shaped curve fitted to the profile.

The second measure described SI and AP displacements (TumorSI and TumorAP) of the whole tumor volume lying within a slice. For each slice, the tumor was outlined on one frame, and the average pixel intensity within the outline was calculated. In repeat frames, an automated exhaustive search was performed to find the rigid coordinate translation that caused the average intensity level within the shifted contour to best match the average intensity on the first frame. Values of the TumorSI and TumorAP displacements within the slice were taken from these moves.

#### 2.2.3 Normal tissue displacement signals

Slice‐specific signals were also generated from the normal thoracic anatomy. Signals representing displacements of the diaphragm and upper and lower chest wall were determined from frame‐to‐frame changes in the locations at which image intensities along line profiles exceeded threshold levels. The line profiles were constructed similarly to those used for the TumorSURF measure. For the diaphragm signal, DIA, the line ran SI through the apex of the diaphragm dome in the slice. For the upper and lower chest wall signals, upper chest wall (UCW) and lower chest wall (LCW), lines ran AP through the sternal angle and top of the diaphragm respectively. The threshold level for DIA was defined as the 50% point on an s‐shaped curve fitted to the intensity profile. For UCW and LCW, the threshold level was typically set around three times the background noise level. A signal representing frame‐to‐frame changes in slice body area, BA, was also produced, using methods described by Liu et al.^21^

#### 2.2.4 Correlative analysis and statistical testing

For individual slices through the tumor center and periphery in each patient's sagittal 4D‐MRI scan, correlations between respiratory signals and tumor displacement were determined across the 25 repeat frames collected for the slice. Correlation strengths were characterized using Pearson's *r* measure. Significances of signal‐to‐signal differences in patient‐by‐patient distributions of *r* values were assessed using the two‐sided Wilcoxon signed rank test.

### 2.3 Slice sorting and tumor volume stitching

#### 2.3.1 Slice‐to‐slice signal standardization

To merge slice‐specific signals into a single signal consistently related to respiratory phase, raw signal values were standardized to provide a *Z*‐score via

(1) $${\mathrm{Standardized}\;\mathrm{signal}}_{m,n}=\left({\mathrm{Raw}\;\mathrm{signal}}_{m,n}-{\bar{S}}_{m}\right)/\sigma_{m}.$$

where *n* denotes a repeat frame of slice *m*, ${\bar{S}}_{m}$ is the average raw signal value for slice *m*, and $\sigma_{m}$ is the standard deviation of signal values for that slice. Additionally, for TumorPC1 the slice‐specific signs of signal values were chosen to consistently correspond with the direction of diaphragm motion.^22^

For the diaphragm signal, two variants of the standardization approach were tested. The first proceeded as in Equation (1) and produced a signal we term DIAss. The second produced a signal termed DIAcc and proceeded largely as in Equation (1), except that the average diaphragm heights and their ranges obtained from repeat frames of the sagittal slices were replaced with measurements made in 25 repeat frames of a single coronal slice. This single coronal slice was collected as part of the coronal 4D‐MRI dataset described in Section 2.1, and was the slice lying at the AP coordinate at which diaphragm heights were measured in the sagittal slices to form the raw diaphragm signal. To generate values of ${\bar{S}}_{m}$ and $\sigma_{m}$ for sagittal slice *m* from the coronal slice, repeat measurements were made of the diaphragm height in the coronal slice at the LR coordinate corresponding to sagittal slice *m* (Figure S1).

This alternative standardization, DIAcc, was motivated by the expectation that average signal values taken from the single coronal slice would describe the underlying LR shape of the diaphragm and ranges of motion along it, whereas the average values in repeat frames of different sagittal slices would reflect the LR shape and motion ranges plus random variations. These random variations arise because the sets of frames collected for any two different sagittal slices during the cycling sequential MRI acquisition will correspond to slightly different sets of respiratory phases.

#### 2.3.2 Frame sorting and image stack formation

For each patient, a 3D image stack was compiled by stitching together 2D slices using the TumorPC1 signal. A set of frames was selected one per slice from the patient's 4D‐MRI scan, the frames being chosen to have the lowest variation in standardized values achievable for any such set of frames (Figure S2). Three further 3D image stacks were similarly compiled for each patient using the UCW, DIAss, and DIAcc signals, selected according to the results of the correlative analysis presented in Section 3.2. For comparison, an additional unsorted stack was built, comprising the first frame collected for each slice of the 4D‐MRI scan.

### 2.4 Tumor volume analysis

#### 2.4.1 Surface roughness

The presence of stitching artifacts was assessed using a quantitative measure of the roughness of superior tumor surface, *Rg*, which is expected to rise as stitching artifacts become more prevalent. A 2nd‐degree polynomial was fitted to heights *h_n_* of the tumor surface determined at *N* points across the surface from SI intensity line profiles running through them. Then *Rg* was calculated as the residual sum of squared differences between these heights and the fitted heights, *h_n‐fitted_*, normalized to the degrees of freedom *D* of the fit to the surface

(2) $$Rg\;=\sum_{n=1}^{N}{\left(h_{n}-h_{n-fitted}\right)}^{2}/\left(D\right)\;$$

where *D* is equal to *N‐6*.

Although the *Rg* measure will inevitably include some underlying real variation of the tumor surface not described by the smooth polynomial fit, this contribution to *Rg* is much reduced compared to simpler measures such as the variance of the upper tumor surface.

#### 2.4.2 Individual and whole cohort statistical testing

For individual patients, significances of differences between the roughness measures *Rg* obtained for tumor volumes compiled using two different standardized signals *S*_1_ and *S*_2_ were assessed using a two‐sided *F*‐test of the ratio $F_{S_{1},S_{2}}$ equal to $Rg_{S_{1}}/Rg_{S_{2}}$.

For the whole cohort, significances of the *F*‐test ratios $F_{S_{1},S_{2}}$ for pairs of signals averaged across all 10 patients were tested using a numerical permutation test. First, the mean of the $F_{S_{1},S_{2}}$ ratios in the 10 patients, weighted by their inverse variances, was calculated as

(3) $${\bar{F}}_{S_{1},S_{2}}=\frac{\sum_{p=1}^{10}w_{p\;}F_{S_{1},S_{2},p}}{\sum_{p=1}^{10}w_{p}}\;$$

where

(4) $$w_{p}={\left\{F_{S_{1},S_{2},p}\left(\frac{1}{D_{1p}}+\frac{1}{D_{2p}}\right)\right\}}^{-1}$$

and *p* denotes the *p*th patient. Then permuted datasets were created in which the real *F*‐statistic $F_{S_{1},S_{2},p}$ obtained for each patient was either retained or replaced with a 50% probability by $1/F_{S_{1},S_{2},p}$. Since the dataset comprised 10 patients, 1024 unique permuted datasets were obtained and an ${\bar{F}}_{S_{1},S_{2}}$ value was calculated for each. The 2‐sided significance of the real ${\bar{F}}_{S_{1},S_{2}}$ value was determined by comparison with the distribution of values in the permuted datasets.

## 3 RESULTS

### 3.1 Tumor characteristics

Table 1 lists tumor locations, clinical stages, and peak‐to‐peak amplitudes of SI and AP tumor motion for the 10 patients studied. There was an even split of left‐ and right‐sided tumors, with nine tumors in the lower lung lobes and one in an upper lobe. Average peak‐to‐peak motion amplitudes across all 10 patients in the tumor center and periphery slices were 10.1 mm SI (range 1.8−22.8 mm) and 6.3 mm AP (range 2.9−15.7 mm).

**TABLE 1:** Tumor characteristics.

Patient	Peak‐to‐peak motion amplitude (mm)		TNM	Stage^a^	Tumor location	
	AP	SI			Laterality	lobe
1	3.8	1.8	T3 N1 M0	IIIA	L	Upper
2	2.9	10.5	T3 N0 M0	IIB	L	Lower
3	3.7	2.7	T2b N2 M0	IIIA	R	Lower
4	2.5	5.5	T4 N0 M0	IIIA	R	Lower
5	9.5	10.0	T1c N0 M0	IA	L	Lower
6	15.7	22.8	T1a N0 M0	IA	R	Lower
7	9.7	13.8	T2a N0 M0	IB	R	Lower
8	4.9	8.1	T1b N0 M0	IA	R	Lower
9	4.8	4.7	T1a N0 M0	IA	L	Lower
10	5.7	21.1	T1b N0 M0	IA	L	Lower

^a^ Staging information follows AJCC 8th edition.^29^

### 3.2 Correlations between signals and tumor displacement

Table 2 lists median Pearson *r* coefficients of correlation between the various signals and tumor displacement measures in slices passing through the centers of the 10 patients’ tumors. Ranges of the *r* values obtained for the 10 patients are listed in Table S1. The median *r* value was 0.89 for correlations between the TumorSI and TumorSURF displacement measures, compared to 0.49 between TumorSI and TumorAP.

**TABLE 2:** Median Pearson *r* coefficients of correlations in tumor central slices between tumor displacement measures and signals.

	Tumor								
Signal	SURF	SI	AP	PC1	PC2	PC3	DIA	UCW	LCW
TumorSI	0.89								
TumorAP	0.40	0.49							
TumorPC1	0.86	0.84	0.58						
TumorPC2	0.12	0.15	0.08	0.00					
TumorPC3	0.16	0.11	0.20	0.00	0.00				
DIA	0.72	0.74	0.40	0.92	0.19	0.12			
UCW	0.45	0.50	0.22	0.63	0.21	0.21	0.50		
LCW	0.52	0.48	0.35	0.62	0.23	0.21	0.56	0.79	
BA	0.48	0.45	0.25	0.52	0.21	0.22	0.45	0.84	0.80

Abbreviations: BA, slice body area signal; DIA, diaphragm signal; LCW, lower chest wall signal; TumorAP, anteroposterior tumor displacement; TumorPC1/2/3, signals of first, second and third principal components of pixel intensities in tumor ROI; TumorSI, superior‐inferior tumor displacement; TumorSURF, tumor superior surface displacement; UCW, upper chest wall signal.

For the TumorPC1 signal, median *r* coefficients of correlation with the TumorSURF, TumorSI, and TumorAP displacement measures were 0.86, 0.84, and 0.58, respectively, higher than for any other signal. For the DIA signal, median *r* coefficients of correlation with the three tumor displacement measures were 0.72, 0.74, and 0.40, moderately larger than for signals based on chest wall and body area. For TumorPC2 and TumorPC3, correlations with the tumor displacement measures were much weaker, with median *r* values ≤0.20. The strengths of correlations with superior‐inferior tumor displacement measures were significantly higher for TumorPC1 than for any other signal (*p* < 0.03) except DIA.

Results in tumor peripheral slices are listed in Table S2 and follow a similar pattern. The median *r* value for correlations between TumorSI and TumorSURF was 0.83, compared to 0.58 for correlations between TumorSI and TumorAP. For the TumorPC1 signal, median *r* coefficients of correlation with TumorSURF and TumorSI were 0.79 and 0.86, respectively. For the DIA signal, correlations with TumorSI and TumorAP had median *r* values of 0.75 and 0.73. For other normal tissue signals, the highest median *r* value of correlations with tumor displacement was 0.47. Strengths of correlations with superior‐inferior tumor displacement measures were significantly higher for TumorPC1 than for other signals (*p* < 0.04) except BA (*p* < 0.06) and DIA.

### 3.3 Tumor surface roughness

**FIGURE 2:**
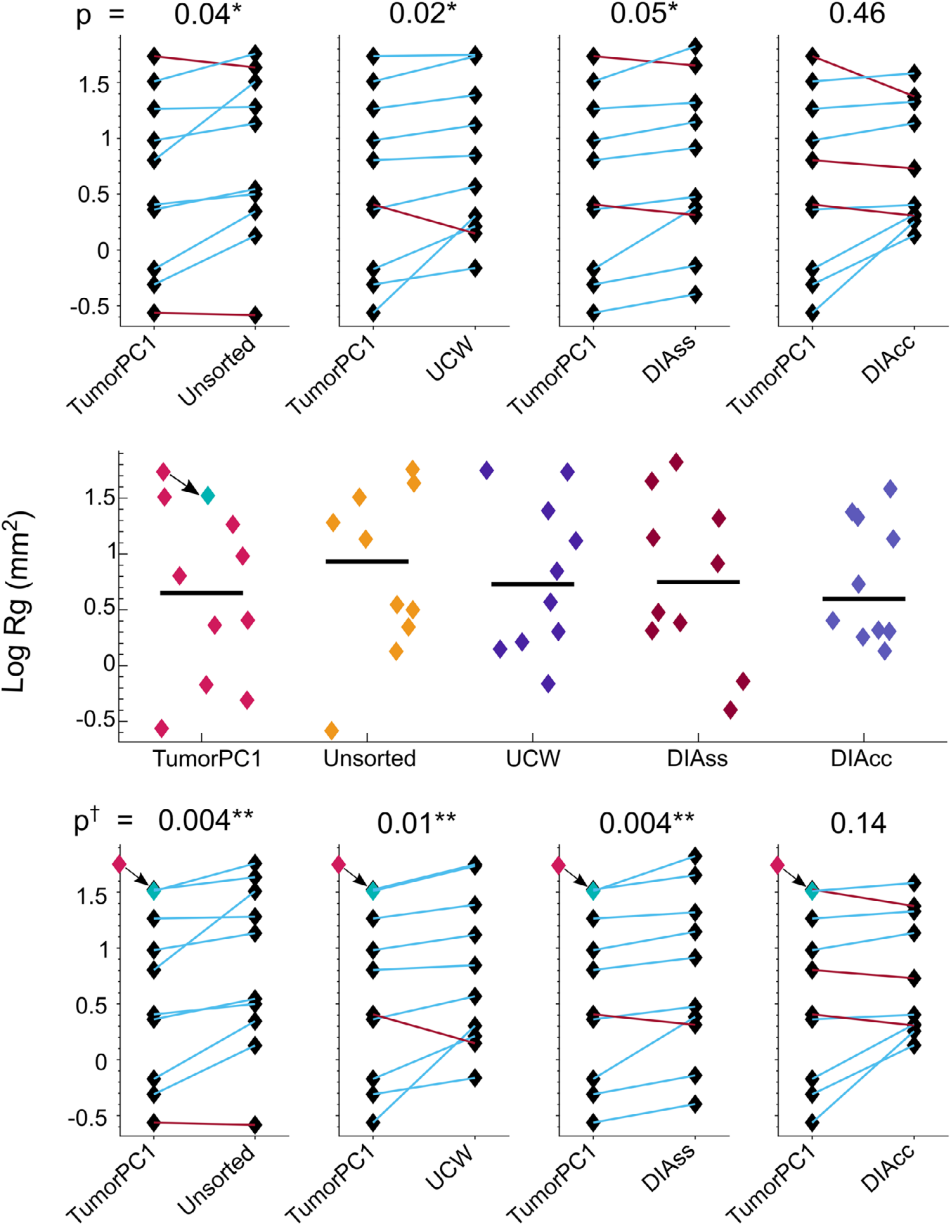
Top row: Paired differences in *Rg* between TumorPC1 and other signals. p‐values from numerical permutation testing of weighted mean *F*‐ratios of *Rg* values are shown above the plot. Middle row: Roughness values *Rg* (mm^2^) of superior tumor surfaces in slice stacks compiled using different signals, with black lines representing median values. The blue data point in the TumorPC1 plot represents the log *Rg* value generated for Patient 2 when the TumorPC1 signal is modified to account for image artefacts (see discussion). Bottom row: Paired differences in *Rg* between TumorPC1 and other signals, reflecting the lower *Rg* of the stack compiled with TumorPC1 for Patient 2 when image artefacts are accounted for. *p*‐Values from numerical permutation testing of modified weighted mean *F*‐ratios of these *Rg* values are shown above the plot. Key. Stacks compiled according to the self‐sorting signals: TumorPC1—1st principal component of tumor ROI; Unsorted—first frame collected for each slice of the 4D‐MRI scan; UCW—upper chest wall; DIAss, DIAcc—diaphragm signals standardized using mean and standard deviation values obtained from sagittal or coronal scan data; ^†^—TumorPC1 signal for Patient 2 is modified to account for image artefacts. ^*^ Significance from permutation testing of weighted mean *Rg F‐*ratios favors the TumorPC1 slice stacks (*p* < 0.05). ^**^Significance favors the TumorPC1 slice stacks (*p* < 0.01).

**FIGURE 3:**
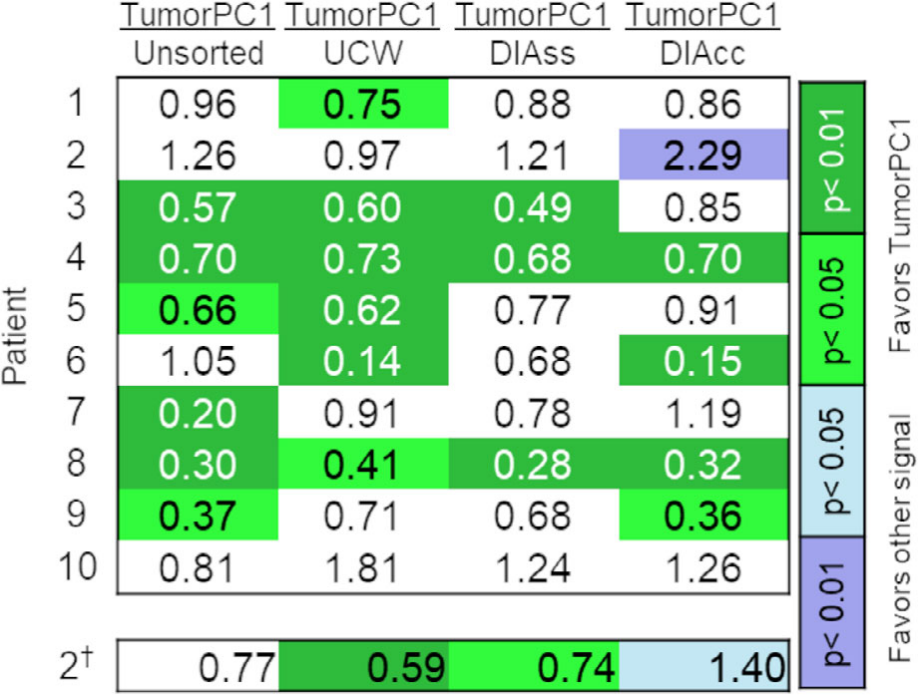
Patient‐by‐patient *F*‐ratios of roughness values *Rg* obtained for tumor surfaces in pairs of image stacks compiled using different signals. Associated significances of the *F*‐ratios are indicated by the color scale, white denoting insignificant *F*‐ratios. Key. Stacks compiled according to the self‐sorting signals: TumorPC1 —1st principal component of tumor ROI; Unsorted—first frame collected for each slice of the 4D‐MRI scan; UCW—upper chest wall; DIAss, DIAcc—diaphragm signals standardized using mean and standard deviation values obtained from sagittal or coronal scan data. ^†^ *—F*‐ratios of *Rg* values generated for Patient 2 when the TumorPC1 signal is modified to account for image artefacts (see discussion).

*Rg* values are listed for the 10 patients in Table 3 and plotted in Figure 2. For each patient, Figure 3 shows *F*‐ratios describing the *Rg* value of tumor surface roughness in the stack compiled using TumorPC1 divided by *Rg* values in stacks compiled using the UCW, DIAss, and DIAcc signals and in the unsorted stack. Associated significance levels are indicated by the color scale. In 32 of 40 comparisons, *Rg* values were lower (better) for stacks computed using TumorPC1 than for stacks compiled using the other signals or in unsorted stacks. In 19 of these comparisons, the difference in performance reached significance (*p* < 0.05 for 5 comparisons, *p* < 0.01 for 14). In 8 of the 40 comparisons *Rg* values were higher (worse) for stacks compiled using TumorPC1 than for stacks compiled using other signals, but the difference was significant (*p* < 0.01) for only one comparison, TumorPC1 versus DIAcc, in only one patient.

**TABLE 3:** Tumor surface roughness values *Rg* (mm^2^) in image stacks compiled using self‐sorted signals.

Patient *(D)*	TumorPC1	Unsorted	UCW	DIAss	DIAcc
1 (*246*)	18.4	19.1	24.4	20.9	21.3
2 (*169*)	54.4	43.2	56.0	44.9	23.8
3 (*150*)	32.4	57.3	54.3	66.6	38.3
4 (*870*)	9.6	13.6	13.1	14.0	13.7
5 (*105*)	2.3	3.5	3.7	3.0	2.5
6 (*21*)	0.3	0.3	2.0	0.4	1.8
7 (*50*)	6.4	32.3	7.0	8.2	5.4
8 *(22*)	0.7	2.2	1.6	2.4	2.1
9 (*18*)	0.5	1.3	0.7	0.7	1.4
10 (*28*)	2.6	3.2	1.4	2.1	2.0
Median	4.5	8.6	5.4	5.6	4.0
2^†^	33.3	43.2	56.0	44.9	23.8

Abbreviations: *D*, degrees of freedom for TumorPC1 surface; DIAss and DIAcc, diaphragm signals standardized using mean and standard deviation values obtained from sagittal or coronal scan data; TumorPC1, first principal component of tumor ROI; UCW, upper chest wall; unsorted, first frame collected for each slice of the 4D‐MRI scan.

^†^ *Rg* value generated for Patient 2 if TumorPC1 signal is modified to account for image artefacts.

Results of whole cohort permutation tests of *F*‐ratios of surface roughness values *Rg* calculated for tumor volumes compiled using the various signals are shown in Table 4. For image stacks compiled using TumorPC1 versus unsorted stacks and stacks compiled using UCW, DIAss, and DIAcc, the weighted means of the *F*‐ratios across all 10 patients were 0.67, 0.69, 0.71, and 0.76, all in favor of TumorPC1. The mean *F*‐ratio differed significantly from one for stacks sorted using TumorPC1 versus unsorted stacks (*p* = 0.04) and versus stacks sorted using UCW (*p* = 0.02) and DIAss (*p* = 0.05). Differences in weighted mean *F*‐ratios between other pairs of signals did not reach significance.

**TABLE 4:** Permutation testing of mean *F*‐ratios between stacks compiled with TumorPC1 versus other self‐sorted signals.

			Null distribution percentile range			
TumorPC1 versus	Weighted mean of *F*‐statistic	*F*‐statistic favors TumorPC1?	2.5th	97.5th	Percentile	*p*‐Value
Unsorted	0.67	Yes	0.67	1.30	2.0	0.04
UCW	0.69	Yes	0.71	1.28	0.9	0.02
DIAss	0.71	Yes	0.71	1.32	2.4	0.05
DIAcc	0.76	Yes	0.68	1.30	23.0	0.46

Abbreviations: DIAss and DIAcc, diaphragm signals standardized using mean and standard deviation values obtained from sagittal or coronal scan data; TumorPC1, first principal component of tumor ROI; UCW, upper chest wall; unsorted, first frame collected for each slice of the 4D‐MRI scan.

**FIGURE 4:**
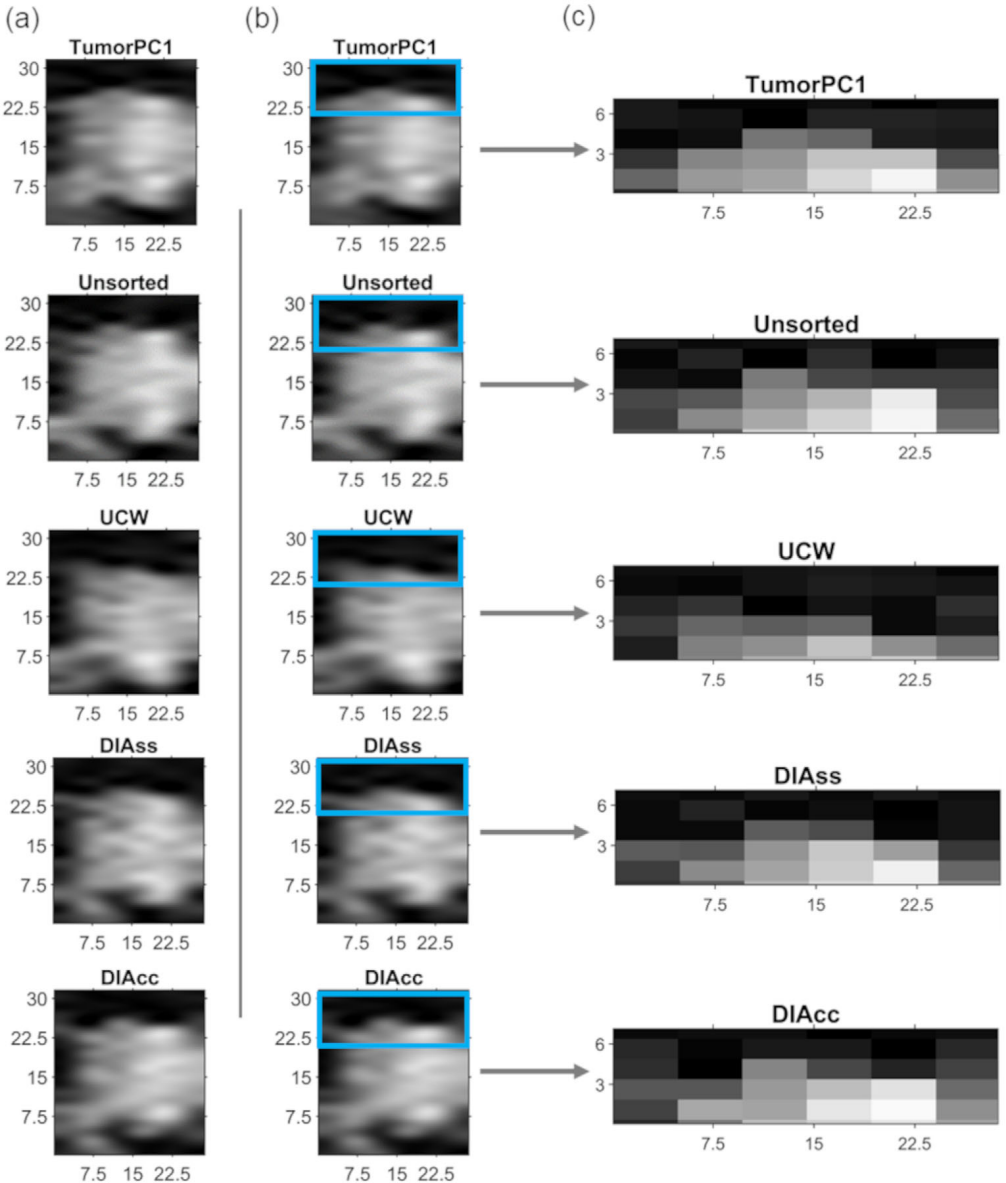
Comparisons for Patient 5 between tumor volumes in stacks of sagittal slices compiled using four signals and in an unsorted stack. The images show the same coronal cut through each of the stacks, with scales shown in mm on the *x*‐ and *y*‐axes. (a) Views of the tumor in a coronal cut through the stacks. (b) Superior surface of the tumor highlighted by a blue box. (c) Zoomed views of the superior surface of the tumor in stacks sorted using the different signals and in the unsorted stack. The surface is smoothest in the stack compiled using TumorPC1.

Coronal cuts through the tumor center are shown in Figure 4 for slice stacks compiled from the 4D‐MRI scan of Patient 5 using the various signals and for an unsorted stack. In the zoomed region, the image can be seen to be smoothest in the stack compiled using TumorPC1.

## 4 DISCUSSION

We have compared the relative abilities of several respiratory signals to stitch together respiratory‐correlated 3D volumes of lung tumors from sagittal 4D‐MRI data. Our comparison included a novel signal based on the first PC of pixel intensity variation in the vicinity of the tumor, TumorPC1.

In image slices through tumor centers and peripheries, median Pearson coefficients of correlation with SI tumor motion were greater for TumorPC1 than for the other signals. Indeed, the strengths of correlations with tumor motion were significantly greater for the TumorPC1 signals than for the CW and BA signals, or for signals derived from the second and third tumor PCs. After TumorPC1, tumor displacement was correlated next most strongly with the DIA signal. These two signals were therefore studied further, along with UCW which was correlated with tumor motion about as strongly as the LCW and BA signals and was obtained from a chest wall region more distant from the diaphragm than was LCW.

The tumor surface roughness measure, *Rag*, was used to compare levels of stitching artifacts in image stacks compiled with the TumorPC1, UCW, DIAss, and DIAcc signals. Across the patient cohort, weighted means of the ratios between *Rg* in stacks compiled using TumorPC1 and *Rg* in stacks compiled using UCW, DIAss, or DIAcc or in unsorted stacks were typically around 0.7, favoring TumorPC1. These ratios differed significantly from one for TumorPC1 versus UCW, DIAss, and unsorted stacks.

Previously, PC analysis has been used to obtain a surrogate signal for motion of the diaphragm but not the tumor itself. Uh et al. (2016) performed a PC analysis on whole repeat frames taken from a synthetic 4D‐MRI scan of a digital thorax phantom.^22^ The authors reported that a self‐sorting signal generated from the first PC of whole frames largely represented diaphragm motion and was relatively insensitive to other independent movements within the thorax, such as tumor motion. Tumor volumes compiled using such a signal based on a PC analysis of whole frames rather than the tumor neighborhood might therefore be expected to be similar to volumes compiled using the diaphragm signal DIAss, which we have found to be rougher than tumor volumes compiled using TumorPC1.

Few studies have used descriptive statistics to measure stitching artifacts within tumor volumes, and to our knowledge, this is the first study to do so using a surface roughness measure. A number of roughness measures have been proposed in the literature.^23^, ^24^ Ours is relatively simple, based on the residual sum of squared differences between the measured surface and a smooth second‐degree polynomial surface fitted to the measured data.

Previously, Paganelli et al. used an electronic phantom to compare lung tumor volumes stitched together according to signals derived from normal tissue displacement measured on phantom images, for example, diaphragm displacement and body area.^14^ However, these authors did not investigate signals obtained from tumor‐specific data. Dice coefficients of similarity between the ground‐truth digital tumor volume and volumes in the slice stacks sorted using the normal tissue‐based signals did not differ significantly from one signal to another. This is consistent with our study, in which tumor surface roughness did not differ significantly between image stacks compiled with different normal tissue signals, but did differ significantly between stacks compiled using TumorPC1 versus those compiled with UCW and DIAss signals.

Little has previously been reported concerning the effects of slice‐to‐slice standardization on the precision of self‐sorting signals. Van de Lindt et al. generated a signal similar to DIAss and found it correlated well with a signal obtained from a diaphragm navigator in a single fiducial slice imaged frequently throughout 4D‐MRI scanning.^17^ But these investigators did not assess tumor volume stitching artifacts or explore other standardizations of the self‐sorting signal. We devised an alternative standardization process leading to a signal, DIAcc, that performed better than DIAss in whole cohort permutation testing of *Rg* ratios, although differences between the two did not reach significance.

DIAcc aimed to eliminate possible inconsistencies in standardization caused by random fluctuations in the sets of respiratory phases at which each sagittal slice was imaged. Although there is some evidence this may have been achieved, the DIAcc standardization requires additional coronal data whereas DIAss can be obtained from sagittal 4D‐MRI data alone. Furthermore, if mean diaphragm shapes and motion ranges change between the times at which the 4D sagittal and coronal data are collected, this will introduce additional uncertainties specific to the DIAcc standardization process. This may explain why tumor surfaces in image stacks compiled using DIAcc were significantly smoother than surfaces compiled using TumorPC1 in just one patient comparison, but were significantly rougher in four comparisons.

For Patient 2, tumor roughness *Rg* in the image stack compiled using DIAcc was notably low relative to *Rg* in stacks compiled with other signals. For the same patient, however, *Rg* in the stack compiled using TumorPC1 was high. The high *Rg* value in the stack compiled using TumorPC1 can be attributed to the inclusion of image artifacts in the cropped area used for the PC analysis. For this patient, it was not possible to crop all slices to include the full extent of the tumor and its motion without also including one of the great vessels within some of the cropped slices. Image artifacts within this vessel were substantial and affected the accuracy of the resultant TumorPC1 signal. We quantified this effect by compiling a new TumorPC1 stack for Patient 2, using modified signal values obtained for slices in which the image artifact was apparent (see Figure S3). The modified values for these slices were generated by masking the blood vessels during the PC analysis. The process of masking artifacts was performed manually, but a similar outcome may be achieved using an automated technique to segment the great blood vessels.^25^

Table 2 shows that using the modified TumorPC1 signal values to compute an image stack for Patient 2 led to a reduced *Rg* value of 33.3 mm^2^ compared to the original value of 54.4 mm^2^. After modification, ratios of *Rg* for Patient 2 significantly favored the stack compiled using TumorPC1 compared to stacks compiled using UCW and DIAss (Figure 3). Mean weighted *F*‐ratios of *Rg* were reduced to 0.65, 0.66, 0.68, and 0.74, favoring stacks compiled with TumorPC1 to unsorted stacks, and stacks compiled with UCW, DIAss, and DIAcc respectively. In permutation testing, the mean weighted ratios of *Rg* for stacks sorted using TumorPC1 versus UCW, DIAss, and unsorted stacks differed from one with an increased significance (Figure 2). The difference in mean weighted *F*‐ratio between stacks compiled with TumorPC1 versus DIAcc did not reach significance, but its *p*‐value was reduced from 0.46 to 0.14.

The image stacks evaluated in this study were built from frames compiled on the basis of respiratory signal values alone. However, frames are commonly sorted by both signal values and their direction of change, thus creating separate sets of inhalation and exhalation phases. This additional split can account for hysteresis effects in which the 3D locations of structures such as tumors depend on both the value of a respiration signal and its recent history, specifically whether it is rising or falling.^26^‐^28^

Such hysteresis effects are typically small, <1 mm.^26^, ^27^ To test whether they might affect our results, we constructed inspiration‐ and expiration‐only stacks based on the TumorPC1, DIAss, and DIAcc signals for four patients with large AP displacements, and calculated *Rg* values for the tumor surfaces in these stacks *(Supplementary data)*. Because 25 repeat frames were collected for each slice, but only around half this number were collected for inhale or exhale frames considered separately, the signal ranges of frames contributing to stacks compiled from inhale‐only or exhale‐only data were consistently wider than the ranges for stacks compiled from the whole dataset. Perhaps as a result, there was no systematic trend for the inhale or exhale tumor surfaces to be less rough than surfaces in stacks compiled from the whole dataset.

## 5 CONCLUSION

An automatic method has been developed to generate respiratory signals directly from changes in pixel intensities in the neighborhoods of lung tumors in 4D‐MRI scans, based on principal components. The TumorPC1 signal derived from the first PC was correlated more strongly with tumor SI motion than were signals derived from body area or displacements of the chest wall or diaphragm in repeat frames of individual sagittal 4D‐MRI slices collected for 10 patients. Differences in correlation strengths were significant for TumorPC1 versus the chest wall signals.

Tumor surfaces had lower roughness measures, *Rg*, in 3D image stacks compiled using the standardized TumorPC1 signal than in stacks compiled using the standardized chest wall or diaphragm signals. Differences between distributions of *Rg* values were significant for stacks compiled using TumorPC1 versus those compiled using a standardized diaphragm signal, the chest wall signal, or unsorted stacks. In summary, the TumorPC1 signal was easy to generate and tumor volumes in 3D image stacks compiled using TumorPC1 were smoother than in stacks sorted using other signals.

## AUTHOR CONTRIBUTIONS

All authors contributed substantially to the conception or design of the work; or the acquisition, analysis, or interpretation of data for the work; drafting the work or revising it critically for important intellectual content; and final approval of the version to be published. All agree to be accountable for all aspects of the work in ensuring that questions related to the accuracy or integrity of any part of the work are appropriately investigated and resolved.

## CONFLICT OF INTEREST STATEMENT

Jamie McClelland has received funding from Elekta unrelated to this research.

## Supporting information

Supporting Information
