# Supplementary material for: Sorting lung tumor volumes from 4D‐MRI data using an automatic tumor‐based signal reduces stitching artifacts
Source: J Appl Clin Med Phys. 2024 Jan 17;25(4):e14262. doi: 10.1002/acm2.14262 (PMC11005973; doi:10.1002/acm2.14262)
Supplement: Supplementary file 1 — Supporting Information [file ACM2-25-e14262-s001.pdf]

Supplementary material

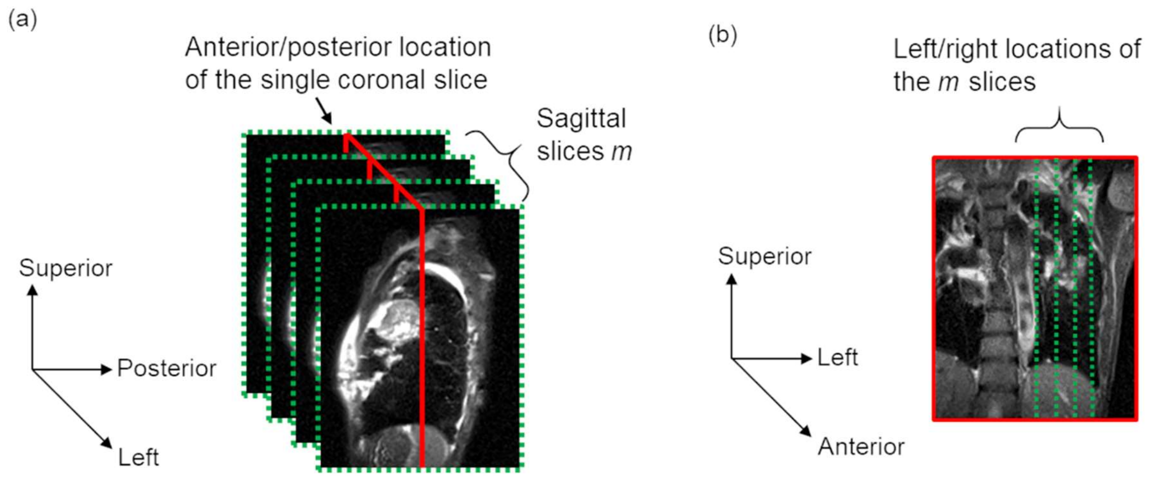

Supplementary figure 1. Selection of the single coronal slice used to obtain additional diaphragm measurements for the DIAcc standardization. a) Schematic view of sagittal slices *m* in which raw signal values are generated. The red line indicates the anterior/posterior location at which diaphragm heights are measured. b) A single coronal slice located at the same anterior/posterior coordinate at which diaphragm heights were originally measured on the sagittal slice. New measurements of diaphragm height are made on this single slice at each left/right location *m* represented schematically by the green dotted lines, the real slice spacing being 0.5 cm apart.

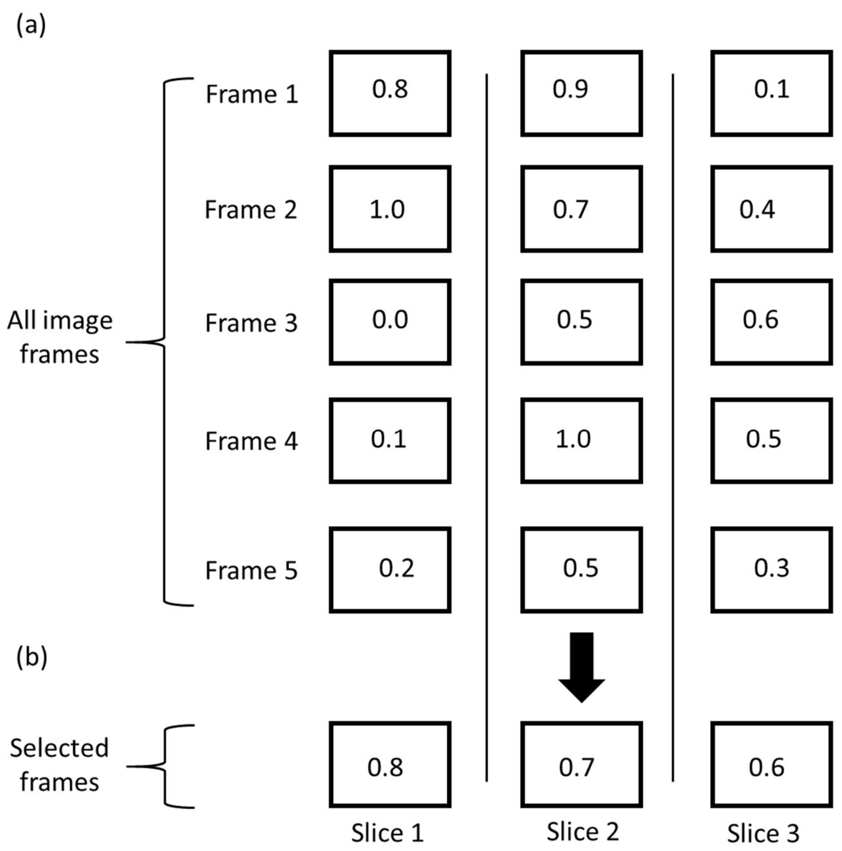

Supplementary figure 2. Schematic representation of the frame selection process. (a) Each image frame is shown with its associated signal z-score. In reality, there are 25 frames per slice and 3-20 tumor slices, and the z-scores would be more evenly distributed within the slices. (b) Frames are selected one-per-slice to have the lowest variation in signature z-score of any such combination. The frames selected in this hypothetical situation have a variation in z-score of 0.2 compared to a variation of 1.0 in the overall dataset.

Supplementary table 1. Medians and ranges (shown in parentheses) of the Pearson coefficients *r* of correlations between tumor displacement and signal measures in tumor central slices.

Signal	TumorSURF	TumorSI	TumorAP	TumorPC1	TumorPC2	TumorPC3	DIA	UCW	LCW
**TumorSI**	0.89 (0.10-1.00)								
**TumorAP**	0.40 (0.00-0.88)	0.49 (0.00-0.90)							
**TumorPC1**	0.86 (0.27-0.98)	0.84 (0.37-0.97)	0.58 (0.00-0.96)						
**TumorPC2**	0.12 (0.01-0.26)	0.15 (0.08-0.80)	0.08 (0.00-0.44)	0.00 (0.00-0.00)					
**TumorPC3**	0.16 (0.00-0.38)	0.11 (0.04-0.39)	0.20 (0.00-0.50)	0.00 (0.00-0.00)	0.00 (0.00-0.00)				
**DIA**	0.72 (0.01-0.98)	0.74 (0.24-0.98)	0.40 (0.00-0.88)	0.92 (0.31-0.97)	0.19 (0.05-0.91)	0.12 (0.02-0.33)			
**UCW**	0.45 (0.01-0.95)	0.50 (0.07-0.95)	0.22 (0.00-0.67)	0.63 (0.19-0.92)	0.21 (0.01-0.69)	0.21 (0.03-0.38)	0.50 (0.15-0.94)		
**LCW**	0.52 (0.14-0.82)	0.48 (0.12-0.80)	0.35 (0.00-0.88)	0.62 (0.20-0.91)	0.23 (0.06-0.66)	0.21 (0.00-0.29)	0.56 (0.13-0.85)	0.79 (0.10-0.95)	
**BA**	0.48 (0.01-0.74)	0.45 (0.17-0.75)	0.25 (0.00-0.81)	0.52 (0.18-0.92)	0.21 (0.05-0.56)	0.22 (0.04-0.40)	0.45 (0.00-0.74)	0.84 (0.35-0.93)	0.80 (0.23-0.96)

Key. TumorSURF – tumor superior surface displacement; TumorSI – superior-inferior tumor displacement; TumorAP – anteroposterior tumor displacement; TumorPC1/2/3 – 1st, 2nd,3rd principal components of pixel intensities in tumor ROI; DIA – diaphragm; UCW – upper chest wall; LCW – lower chest wall; BA – slice body area.

Supplementary table 2. Medians and ranges (shown in parentheses) of Pearson coefficients *r* of correlations between tumor displacement and signal measures in tumor peripheral slices.

Signal	TumorSURF	TumorSI	TumorAP	TumorPC1	TumorPC2	TumorPC3	DIA	UCW	LCW
**TumorSI**	0.83 (0.01-0.98)								
**TumorAP**	0.33 (0.12-0.77)	0.58 (0.04-0.76)							
**TumorPC1**	0.79 (0.21-0.95)	0.86 (0.61-0.97)	0.37 (0.02-0.87)						
**TumorPC2**	0.23 (0.07-0.63)	0.14 (0.03-0.62)	0.31 (0.02-0.59)	0.00 (0.00-0.00)					
**TumorPC3**	0.10 (0.00-0.40)	0.08 (0.01-0.34)	0.24 (0.04-0.49)	0.00 (0.00-0.00)	0.00 (0.00-0.00)				
**DIA**	0.75 (0.35-0.97)	0.73 (0.37-0.99)	0.42 (0.10-0.86)	0.80 (0.37-0.98)	0.17 (0.02-0.81)	0.12 (0.00-0.62)			
**UCW**	0.37 (0.16-0.97)	0.32 (0.08-0.97)	0.44 (0.26-0.72)	0.61 (0.14-0.95)	0.43 (0.01-0.67)	0.24 (0.11-0.59)	0.60 (0.02-0.97)		
**LCW**	0.42 (0.19-0.81)	0.50 (0.23-0.79)	0.47 (0.05-0.85)	0.61 (0.36-0.95)	0.29 (0.01-0.70)	0.23 (0.00-0.56)	0.56 (0.20-0.92)	0.82 (0.00-0.95)	
**BA**	0.44 (0.21-0.91)	0.32 (0.14-0.90)	0.31 (0.01-0.84)	0.52 (0.09-0.87)	0.32 (0.00-0.85)	0.31 (0.01-0.60)	0.55 (0.05-0.95)	0.84 (0.53-0.96)	0.81 (0.42-0.97)

Key. TumorSURF – tumor superior surface displacement; TumorSI – superior-inferior tumor displacement; TumorAP – anteroposterior tumor displacement; TumorPC1/2/3 – 1st, 2nd,3rd principal components of pixel intensities in tumor ROI; DIA – diaphragm; UCW – upper chest wall; LCW – lower chest wall; BA – slice body area.

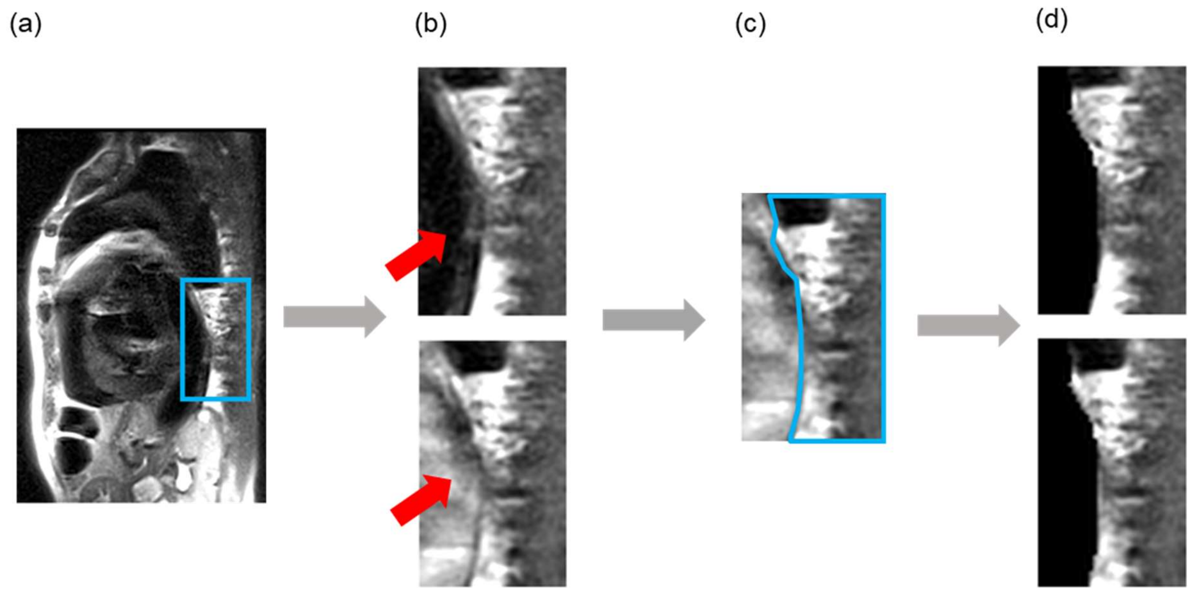

Supplementary figure 3. Creation of a modified TumorPC1 signal for Patient 2 to improve the accuracy of TumorPC1 in the presence of image artifacts in a blood vessel. (a) A sagittal image slice showing the cropping ROI in blue. The ROI is a rectangular shape drawn to encompass the tumor and its extent of motion in all sagittal image slices. All slices are cropped with the same ROI. (b) A closeup of two of the twenty-five frames for this slice, cropped using the rectangular ROI. The red arrows point to a region in a blood vessel where image artefacts caused notable changes in frame-to-frame pixel intensities. (c) A region is manually delineated within the cropped images in order to mask the blood vessel, highlighted in blue. (d) Images of the two frames shown in (b) after the masking. The same masking is applied to all frames of the affected slices. Such masked frames are used to generate TumorPC1.

The effect of hysteresis on tumor surface roughness.

To test the effect of tumor hysteresis on image sorting, four patients with large AP tumor displacements were selected for a sub-analysis. For patients 5, 6, 8 and 10, inspiration and expiration-only stacks were compiled by adding signs to the signal values, indicating positive or negative directions of change over time. Direction was determined by comparing the standardized signal value obtained for a repeat frame of slice m with the standardized signals obtained for the repeat frames of the slices imaged immediately before and after m, namely $m \pm 2$. Using these signed signals, new inhale- and exhale-only image stacks were built by selecting the sets of frames with exclusively one sign or the other which had the minimum variation in absolute signal values of any such set drawn one-per-slice.

For these four patients, Supplementary Table 3 lists roughness values *Rg* of superior tumor surfaces in the stacks compiled from inhale-only, exhale-only and complete data using the TumorPC1, DIAss and DIAcc signals. Ranges of standardized signal values for frames contributing to the compiled stacks are listed in Supplementary Table 4.

For patient 5, the mean diaphragm shape and motion range differed substantially between the sagittal and coronal 4D-MRI datasets. As a result, in one imaged slice of the tumor, all frames were assigned an inspiration sign after standardization. This meant that an exhale stack could not be compiled for the DIAcc signal and was therefore not available for analysis.

Of 23 possible comparisons, only four of the inspiration- or expiration-only stacks compiled using the various signals had lower (better) *Rg* values than stacks compiled from all frames using the TumorPC1 signal. Furthermore, none of these four differences were significant using an F-test. On the other hand, in 19 of the 23

comparisons the stacks compiled from all frames using TumorPC1 had lower (better) *Rg* values than did stacks compiled from inhale- or exhale-only frames using the various signals, and five of these differences reached significance ($p<0.05$).

Supplementary table 3. Roughness measures (*Rg*) of tumor superior surfaces in slice stacks compiled using the TumorPC1, DIAss and DIAcc signals from whole sagittal 4D-MRI datasets or from inspiration- or expiration-only frames.

Pt	Signal	*Rg* (mm^2)		
		**All frames**	**Inspiration**	**Expiration**
5	TumorPC1	2.30	2.29	2.77
	DIAss	3.00	2.13	3.97
	DIAcc	2.90	2.40	NA
6	TumorPC1	0.27	0.57	0.57
	DIAss	0.40	1.57	0.93
	DIAcc	1.81	0.52	1.26
8	TumorPC1	0.67	1.10	0.94
	DIAss	2.42	0.95	0.98
	DIAcc	2.08	1.01	0.98
10	TumorPC1	2.60	2.33	3.45
	DIAss	2.10	3.41	2.65
	DIAcc	2.00	1.85	6.04

Key. Stacks compiled according to the self-sorting signals: TumorPC1 – 1st principal component of tumor ROI; DIAss, DIAcc – diaphragm signals standardized using mean and standard deviation values obtained from sagittal or coronal scan data.

Supplementary table 4. Ranges of standardized signals in image stacks compiled from inhale-only, exhale-only and whole 4D-MRI sagittal slice stacks using the TumorPC1, DIAss and DIAcc signals.

**Pt**	**Signal**	**Signal Range**		
		**All frames**	**Inspiration**	**Expiration**
5	TumorPC1	0.04	0.75	0.08
	DIAss	0.07	0.39	0.13
	DIAcc	0.04	0.24	NA
6	TumorPC1	0.00	0.07	0.09
	DIAss	0.01	0.02	0.26
	DIAcc	0.01	0.20	0.07
8	TumorPC1	0.02	0.11	0.03
	DIAss	0.01	0.05	0.01
	DIAcc	0.01	0.52	0.01
10	TumorPC1	0.03	0.22	0.03
	DIAss	0.01	0.04	0.03
	DIAcc	0.01	0.01	0.08

Key. Stacks compiled according to the self-sorting signals: TumorPC1 – 1st principal component of tumor ROI; DIAss, DIAcc – diaphragm signals standardized using mean and standard deviation values obtained from sagittal or coronal scan data.
